# Highly pathogenic avian influenza H5N1 virus could partly be evacuated by pregnant BALB/c mouse during abortion or preterm delivery

**DOI:** 10.1186/1743-422X-8-342

**Published:** 2011-07-08

**Authors:** Lili Xu, Linlin Bao, Wei Deng, Chuan Qin

**Affiliations:** 1 Institute of Laboratory Animal Sciences, Chinese Academy of Medical Sciences (CAMS) & Comparative Medicine Center, Peking Union Medical Collage (PUMC), Beijing, 100021, China; 2 Key Laboratory of Human Disease Comparative Medicine, Ministry of Health, Beijing, 100021, PR China

## Abstract

The highly pathogenic avian influenza H5N1 virus is one of candidates for future pandemic. Since H5N1 viruses had previously been isolated only from avian species, the outbreak raised questions about the ability of these viruses to cause severe disease and death in humans. Pregnant women are at increased risk for influenza-associated illness and death. However, little is known about whether influenza viruses could transmit to the fetus through the placenta, and the effects of abortion and preterm delivery to maternal influenza infection are not well understood. We found that the H5N1 viruses could vertical transmit to the fetus through the placenta in the BALB/c mouse model, and the viruses could partly be evacuated by the pregnant mice during abortion or preterm delivery. This study may further our understanding about the transmission of this highly pathogenic avian influenza viruses, supply optimized clinical treatment method for pregnant women, and shed some light on better preventing and controlling for future potential outbreak of H5N1 influenza pandemic.

## Findings

Influenza A viruses belong to Orthomyxoviridae and consist of segmented, negative-sense RNA genomes. The known subtypes of influenza A viruses include 16 subtypes of hemagglutinin (HA) and nine subtypes of neuraminidase (NA) [1]. During 1997 in Hong Kong, 18 human cases of respiratory illness, including 6 fatalities, were caused by highly pathogenic avian influenza H5N1 viruses [2-4]. The H5N1 infections in humans were preceded by the circulation of H5N1 viruses in birds, first in poultry farms and later in wholesale and retail poultry markets in Hong Kong [5]. Concerns that the virus might acquire the ability to efficiently spread between humans have led public health authorities to accelerate preparations for pandemic influenza. The fact that the H5N1 viruses resulted in severe or fatal respiratory disease in the majority of infected persons aged 13 to 60 years was of particular concern since this age group is not normally considered to be at increased risk for death and complications from influenza [6,7].

Pregnant women are at high risk for severe complications of influenza during previous pandemics of 1918 and 1957 [8-10]. Among 1,350 reported cases of influenza among pregnant women during the pandemic of 1918, the proportion of deaths was reported to be 27% [9]. Similarly, among a small case series of 86 pregnant women hospitalized in Chicago for influenza in 1918, 45% died [11]. Among pregnancy-associated deaths in Minnesota during the 1957 pandemic, influenza was the leading cause of death, accounting for nearly 20% of deaths associated with pregnancy during the pandemic period, half of women of reproductive age who died were pregnant [8]. For the highly pathogenic avian influenza H5N1 virus, a previous healthy pregnant woman at 4 months' gestation died in Anhui Province of China in 2005 since of involved in slaughtering and defeathering sick poultry infected with H5N1 [12]. Furthermore, pregnancy has also been a risk factor for increased illness during interpandemic periods [13]. The increased morbility and mortality are believed to be related to several physiologic changes that occur during pregnancy. Because of mechanical and hormonal alterations that occur during pregnancy, several changes also occur to the cardiovascular and respiratory systems, including increased heart rate, stroke volume, oxygen consumption, and decreased lung capacity [14]. Relevant immunologic alterations also occur during pregnancy, with a shift away from cell-mediated immunity toward humoral immunity. This shift can render pregnant women more susceptible to, or more severely affected by, certain viral pathogens, including influenza [15].

Although certain infections are well recognized to increase the risk for influenza-associated illness and death for pregnant women, the effects of abortion and preterm delivery to maternal influenza infection are not well understood. To address this problem, mouse model for H5N1 virus infection was established primarily. Briefly, female 5-week-old SPF level BALB/c mice (Institute of Laboratory Animal Sciences, Beijing) were used. Mice were anesthetized and inoculated intranasally with 10^2 ^TCID_50 _of A/Shenzhen/406H/2006 virus strain in a volume of 50 μl, and were monitored for clinical signs over a 14 day period. Meanwhile, additional inoculated mice were euthanized at 5 days post infection (d.p.i) for assessment of infectious virus in tissues. The procedures were approved by the Institute of Animal Use and Care Committee of the Institute of Laboratory Animal Science, Peking Union Medical College (ILAS-PC-2010-006) and all experiments involving the H5N1 virus were conducted under biosafety level 3 (ABSL-3) conditions. Two days post challenge, typical clinical symptoms including hunched posture, ruffled fur, loss of appetite began to exhibit. Mice began to die at 5 d.p.i, and all mice died within 8 d.p.i. High titers (TCID_50 _> 10^6^) of infectious virus could be detected in lung tissues of infected mice which euthanized at 5 d.p.i, and the RNA copy numbers reached 10^6 ^per milligram.

Based on this mouse model, two independent experiments were carried out to verify whether the highly pathogenic avian influenza H5N1 viruses could vertical transmit to the fetus through the placenta, and the viruses may partly be evacuated by the pregnant women during abortion or preterm delivery.

**Figure 1:**
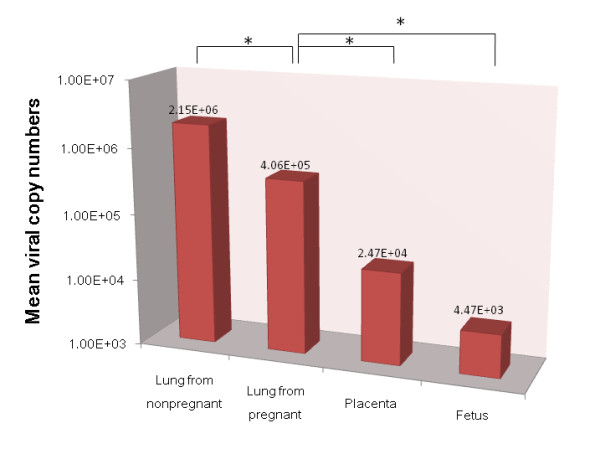
**The RNA loads of H5N1 viruses in lung tissues from nonpregnant mice, and lung tissues, placenta, and fetus from pregnant mice which euthanized at 5 days post infection**. Data represented the mean viral RNA load per microgram ± standard deviation.* represent statistical significance at *P*< 0.05 (one-way ANOVA).

Firstly, five mice in the second trimesters of pregnancy were intranasally challenged with H5N1 virus. Meanwhile, another five nonpregnant mice with the same age were used as control. Five days post challenge, mice were euthanized and lung and placenta from maternal mice and fetuses were collected separately for viral nucleotide material quantification. Results demonstrated that the mean viral nucleotide copy numbers of lung tissues from pregnant mice were significant lower than those from nonpregnant mice (*P*< 0.05). Meanwhile, viral nucleotides could also been detected in the placenta and fetus (Figure 1), although the copy numbers were significant lower than those from maternal lung tissues (*P*< 0.05). All these data verified that H5N1 virus could vertical transmit to the fetus through the placenta, and the virus load in maternal mice could partly be evacuated during abortion.

**Figure 2:**
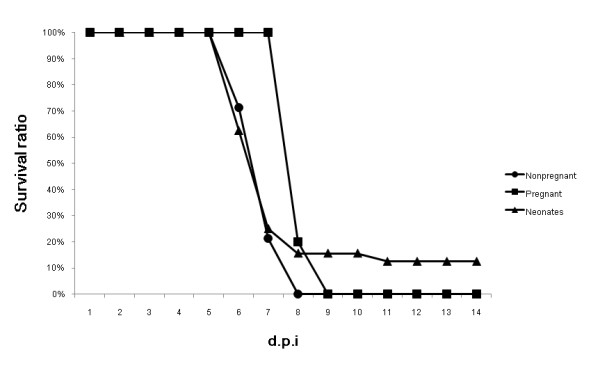
**Survival percentage of nonpregnant and pregnant mice which challenged with H5N1 viruses and neonates up to 14 days post inoculation**.

**Figure 3:**
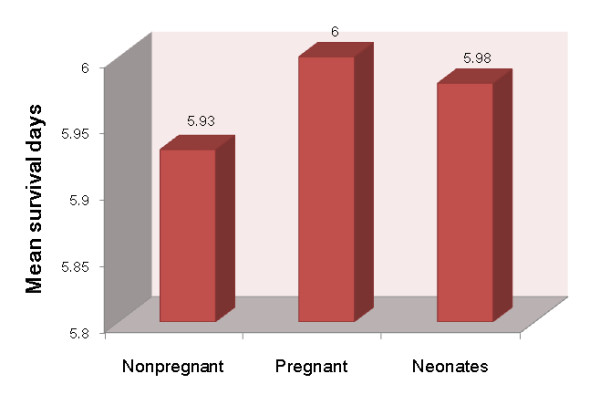
**The mean survival days of nonpregnant, pregnant, and infant mice which challenged with H5N1 viruses**.

Secondly, eight mice in the third trimesters of pregnancy were intranasally challenged with H5N1 virus, and each pregnant mouse delivered 5 to 7 neonates the next day post infection. Meanwhile, another fourteen nonpregnant mice with the same age were used as control. All mice were monitored daily for mortality up to 14 d.p.i. Results showed that although all adult mice died within 14 days (Figure 2), the mean survival days of pregnant mice were longer compared with those nonpregnant (Figure 3). Meanwhile, results showed that 12.5% neonates survived up to 14 days (Figure 2). However, since the neonates born the next day post the maternal mice were challenged, whether the H5N1 viruses were transmitted to neonates through the placenta, or aerosol, or breastfeeding was unknown and needs further research.

All above data based on mouse model probably proposed that the H5N1 viruses could vertical transmit to the fetus through the placenta, and the viruses may partly be evacuated by the pregnant women during abortion or preterm delivery. However, these primary findings need to be confirmed by further studies, especially clinical cases monitor and observation. If this phenomenon was proven to be similar in human, it may further our understanding about the transmission of this highly pathogenic avian influenza viruses, supply optimized clinical treatment method for pregnant women, and shed some light on better preventing and controlling for future potential outbreak of H5N1 influenza pandemic.

## Competing interests

The authors declare that they have no competing interests.

## Authors' contributions

LLX carried out the animal challenge and observation, real-time PCR, statistical analysis and drafted the manuscript. LLB participated in the animal experiments. WD performed pathological analysis. CQ predicated in the design of the study. All authors read and approved the final manuscript.
